# Updating the Wnt pathways

**DOI:** 10.1042/BSR20140119

**Published:** 2014-10-17

**Authors:** Jia Yu, David M. Virshup

**Affiliations:** *Program in Cancer and Stem Cell Biology, Duke-NUS Graduate Medical School, 8 College Road, Singapore 169857, Singapore; †Institute of Medical Biology, A*STAR, Singapore 138648, Singapore; ‡Department of Biochemistry, National University of Singapore, Singapore 117597, Singapore; §Department of Pediatrics, Duke University, Durham, NC 27710, U.S.A.

**Keywords:** adenomatous polyposis coli, planar cell polarity (PCP), Wnt, APC, adenomatous polyposis coli, BAR, Bin-Amphiphysin-Rvs, CBP, CREB (cAMP response element-binding)-binding protein, COP, coat protein complex, CRD, cysteine-rich domain, CTD, C-terminal domain, CK1α, casein kinase 1 α, ER, endoplasmic reticulum FAP, familial adenomatous polyposis, FDH, focal dermal hypoplasia, GSK3β, glycogen synthase kinase 3β, LEF, lymphoid enhancer-binding factor, LRP, lipoprotein receptor-related protein, NTD, N-terminal domain, PCP, planar cell polarity, PORCN, protein Porcupine, Ror2, receptor tyrosine kinase-like orphan receptor 2, RSPO, R-Spondin, sFRP, secreted Frizzled-related protein, SNX-1, sorting nexin-1, swim, Wingless-interacting molecule, TCF, T cell-specific factor

## Abstract

In the three decades since the discovery of the Wnt1 proto-oncogene in virus-induced mouse mammary tumours, our understanding of the signalling pathways that are regulated by the Wnt proteins has progressively expanded. Wnts are involved in an complex signalling network that governs multiple biological processes and cross-talk with multiple additional signalling cascades, including the Notch, FGF (fibroblast growth factor), SHH (Sonic hedgehog), EGF (epidermal growth factor) and Hippo pathways. The Wnt signalling pathway also illustrates the link between abnormal regulation of the developmental processes and disease manifestation. Here we provide an overview of Wnt-regulated signalling cascades and highlight recent advances. We focus on new findings regarding the dedicated Wnt production and secretion pathway with potential therapeutic targets that might be beneficial for patients with Wnt-related diseases.

## INTRODUCTION

In multicellular organisms, cell–cell communication is critical for development, homoeostasis, and response to injury. Long and short-range signalling molecules (ligands) bind to cellular receptors and convey messages by initiating a cascade of cytoplasmic signalling events. These signals can lead to dramatic changes in cellular protein abundance, localization and activity, as well as the changes in the transcriptome, and ultimately, in the epigenome. All steps in these pathway can potentially be regulated and the intricate interactions among the ligands, receptors, and intracellular signalling molecules add both complexity and robustness into the system.

The Wnt family of signalling ligands and associated downstream mediators play key roles in short range cell–cell signalling within specific tissues that is essential for both developmental and homoeostatic processes. Adding to their importance, dysregulation of Wnt pathways can result in diverse pathologic disorders (recently reviewed by Clevers and Nusse [1]). The classification of Wnt signalling pathways is complex and historical. One of the most intensively studied consequence of Wnt signalling is the stabilization of the multifunctional protein β-catenin, a process that can activate gene transcription and is often referred to as canonical signalling. This Wnt/β-catenin pathway is readily studied by the use of artificial reporter constructs containing synthetic TCF (T cell-specific factor)/LEF (lymphoid enhancer-binding factor) bindings sites, such as TOPFLASH and SuperTOPFLASH [2,3]. The ease of use of these tools has facilitated intense study of this specific subset of the Wnt signalling, perhaps at the expense of other equally important but less easily studied responses. There are diverse additional consequences of Wnt–receptor interactions, such as β-catenin independent regulation of PCP (planar cell polarity) pathway, and these harder-to-study pathways are sometimes lumped into a category referred to non-canonical signalling. However, since there are 19 mammalian Wnt genes, ten Frizzled receptors and additional Wnt receptors and co-activators, the adverb ‘non-canonical’ is not particularly informative. Given the complexity of these pathways, it is preferable to specify as much as possible which downstream pathways are regulated by specific ligands. For example, referring to Wnt/β-catenin signalling, or Wnt/PCP signalling is more informative than a less specific statement about non-canonical Wnt signalling.

## A BRIEF OVERVIEW OF THE Wnt/β-CATENIN SIGNALLING CASCADE

### A simplified view of the transcriptional activation pathway

**Figure 1:**
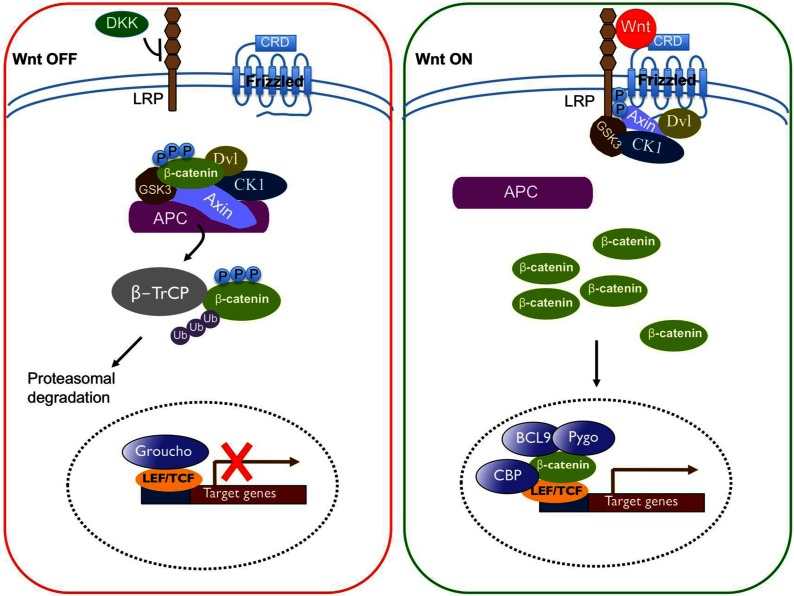
A classical view of the canonical Wnt pathway Refer to the text for detailed explanation

Free cytoplasmic β-catenin is a key player in a major subset of Wnt signalling cascades. When specific Wnt ligands are absent, cytoplasmic β-catenin levels are kept at low via constant targeting by a multiprotein destruction complex, recently comprehensively reviewed by Niehrs [4]. The destruction complex is composed of two scaffold proteins, Axin and APC (adenomatous polyposis coli), which facilitate the phosphorylation of β-catenin by CK1α (casein kinase 1 α) and GSK3β (glycogen synthase kinase 3β). Phosphorylation of β-catenin results in its recognition and ubiquitination by the E3 ubiquitin ligase β-TrCP (β-transducin repeats containing protein), which earmarks β-catenin for proteasomal degradation. Under such conditions, the nuclear transcription factor lymphoid enhancer-binding factor/T cell-specific (LEF/TCF) is associated with Groucho and represses target gene expression [5,6]. (Figure 1, Wnt OFF state).

Conversely, when Wnt ligands bind to Frizzled (Fzd) and its co-receptor, the low-density LRP (lipoprotein receptor-related protein)5/6, the receptors multimerize and form larger multiprotein complexes, which are visible as distinct punctate structures in the cytoplasm [7]. These aggregates, termed signalosomes, are the locations where a cascade of phosphorylation events take place that prevents β-catenin proteasomal degradation [8]. Stabilized β-catenin then accumulates in the cytoplasm. A subset of β-catenin can be translocated into the nucleus via a process that in some cases requires activated Ras signalling [9–11]. Nuclear β-catenin displaces Groucho and forms a complex with the BCL9 (B-cell lymphoma 9 protein), Pygopus, histone modifier CBP [CREB (cAMP response element-binding)-binding protein] as well as tissue-specific transcriptional activators, and converts LEF/TCF from a transcriptional repressor to an activator that turns on gene expression in a very cell-type-specific manner [12,13] (Figure 1, Wnt ON state).

Alternative models for how Wnt/GSK3 signalling regulates cell fate have recently been proposed. The Wnt/LRP6-mediated down-regulation of GSK3 activity can occur through internalization of GSK3 into multivesicular bodies [14–16]. This causes a global decrease in cytosolic GSK3 activity, and hence decreased phosphorylation-mediated degradation of a large number of cellular proteins. This transcription-independent mode of Wnt regulation may be especially important in G2/M phase of the cell cycle when CDK14/Cyclin Y, which phosphorylates LRP6 to drive GSK3 inhibition, is most active [14,17]. This emerging protein stabilization paradigm may also explain why more specific Wnt/β-catenin transcriptional signatures have been elusive and unreliable.

### Updating the destruction complex

Detailed kinetic studies have been carried out in cell culture to investigate how β-catenin levels are regulated after Wnt stimulation [18]. By carefully comparing the changes in differentially phosphorylated β-catenin (i.e. either by GSK3 or CK1α), unphosphorylated, and total β-catenin, the authors conclude that β-catenin degradation is still occurring after long-term Wnt stimulation when a new steady state has been reached. More importantly, Wnt only partially inhibits the function of the destruction complex for both phosphorylation events [18], which is in contrast to the general belief that Wnt ligand binding blocks β-catenin phosphorylation as well as ubiquitination-mediated degradation. Using endogenous Axin immunoprecipitation, it has also been reported that the Axin destruction complex remains intact after Wnt stimulation [19]. Instead of inhibiting phosphorylation, β-catenin ubiquitination is hindered, resulting in the saturation of the destruction complex with phosphorylated β-catenin. In this model, the newly synthesized and accumulating free β-catenin is the key player for the initiation of transcription [19].

The exact mechanisms underlying how the signals perceived by the receptors are transduced to the destruction complex remain controversial. Upon Wnt stimulation, the PPPSP motif at the cytoplasmic tail of LRP6 is phosphorylated, leading to the recruitment of Axin to LRP6 [20]. Multiple kinases can phosphorylate and regulate LRP6 activity, including membrane associated CK1γ (casein kinase 1γ) [20], GSK3 [21], other casein kinase 1 family members [22], PKA (protein kinase A) [23], GRK (G protein-coupled receptor kinase) 5/6 [24], and in mitosis, the cell-cycle-dependent kinase CDK14 with its cyclin, cyclin Y [17,25]. After LRP6 activation, GSK3 and PP1 (protein phosphatase 1) together modulate Axin phosphorylation and hence conformation. This in turn regulates Axin's scaffolding function [26–28]. When Wnt is absent, Axin is phosphorylated by GSK3, resulting an open state of Axin ready for activation. Upon Wnt stimulation, the phosphorylated LRP6 cytoplasmic tail can inhibit GSK3-mediated phosphorylation [29,30]. Thus the balance of Axin phosphorylation shifts to dephosphorylation via the activity of PP1. As a result, Axin is in a closed state via intramolecular interactions, unable to associate with β-catenin and LRP6. The freed phospho-LRP6 is thus able to inactivate other open state and activated phospho-Axin molecules [26]. It should be noted that there are several alternative models that explain the regulation of β-catenin abundance following phosphorylation of LRP6, including axin degradation or sequestration, or displacement of YAP and TAZ [15,19,31–33]. Several other studies suggest that regulation of axin abundance is a critical control point for Wnt/β-catenin signalling [31,34,35]. Different modes of pathway regulation may co-exist within the same cells or tissues, with alternative pathways varying in importance depending on cellular context.

## WNT RECEPTOR COMPLEXITY AND DYNAMICS

### Context-dependent response with other ligands and receptors

The interaction of Wnts with Frizzled and LRP5/6 at the membrane is regulated by a dense network of additional signalling agonists and antagonists. Diverse non-Wnt proteins also bind to the Wnts and to the Frizzled receptors to either activate or inhibit Wnt/β–catenin pathway. For example, Norrie disease protein Norrin binds to Fzd4 receptor with high affinity to activate the Wnt pathway and mutations of either Norrin or Fzd4 lead to incomplete retinal vascularization [36]. Norrin has also recently been proposed to be a ligand for the LGR4 receptor [37]. Several sFRPs (secreted Frizzled-related proteins) are expressed that can competitively bind to Wnt ligands and prevent the interaction between Wnt and its receptors [38]. sFRPs may also bind to Fzd receptor through the dimerization of their homologous CRDs (cysteine-rich domains) [38], and sFRPs can activates Wnt signalling at lower, perhaps more physiological concentrations in certain cellular contexts [39]. Another class of Wnt inhibitors, the DKK (Dickkopf) family of proteins, potently inhibits Wnt/β-catenin signalling by binding to the Wnt co-receptors LRP5/6 [40]. The WISE and Sclerostin (SOST) family of proteins similarly antagonize Wnt signalling by binding to LRP4, 5 and 6 [41–43]. Inherited mutations in several of these components underlie human skeletal disorders including osteoporosis and increased bone mass syndromes (reviewed recently in [44]). Reconstitution and monoclonal antibody studies have demonstrated that different classes of Wnts interacts with distinct propeller regions of LRP6 [45–47]. These findings provide structural and mechanistic insights as to how different Wnts can interact differently with the same sets of receptors.

The Wnt-binding CRD of the frizzled family of Wnt receptors is also found in the single pass Ror2 (receptor tyrosine kinase-like orphan receptor 2). Wnt5a may interact with Ror2 through this domain [48]. Furthermore, another well-characterized Wnt-binding motif, WIF, is present in the atypical receptor tyrosine kinase Ryk [49]. It appears that the specific signalling cascades induced by Wnts are determined by the combination of ligands and specific receptors in a cellular context. This is exemplified by the activation of JNK (c-Jun N-terminal kinase) and Src kinases after binding of Wnt ligands to Ror2 and Ryk receptors, respectively [49].

### Surface Frizzled levels are regulated by post-translational modifications

A complex new mechanism that controls the abundance of Wnt receptors on the cell surface has recently emerged. Ubiquitination of Frizzled receptors induces their endocytosis, thus reducing their surface receptor abundance and decreasing sensitivity to Wnts [50,51]. RNF43 and ZNRF3 are related transmembrane RING domain-containing E3 ubiquitin ligases whose expression is up-regulated by Wnt signalling. They are expressed in diverse tissues, and are co-expressed with Lgr5 in intestinal stem cells [51]. By ubiquitination of a cytosolic domain of Frizzled, they induce their internalization and degradation. Importantly, these ubiquitin ligases are tightly regulated. RSPOs (R-Spondins) are secreted proteins that sensitize cells to Wnts. They do this by complexing with the extracellular domains of both LGR4/5 and RNF43/ZNRF3, leading to decreased activity of the ubiquitin ligase and hence increased Frizzled abundance [50,52–54]. This mechanism is consistent with the observation that RSPOs cooperate with Wnts but on their own do not activate Wnt/β-catenin signalling.

The RSPO/LGR5/RNF43/Frizzled pathway is critical in both normal stem cell maintenance and in cancer. Inactivating mutations in RNF43 and ZNRF3 occur in ~5% of pancreatic, ovarian, gastric and biliary cancers [55–58]. Pancreatic cancer cells with RNF43 mutations have increased Wnt/β-catenin activity and are especially sensitive to Wnt inhibition [59]. RSPOs were known to be important in the *ex vivo* survival and proliferation of intestinal stem cells [60,61]. The RSPO in the intestinal stem cell niche appears to be RSPO3, where it is supplied by the stroma [62]. Notably, RSPO2 and RSPO3 are overexpressed by chromosome translocations in a subset of colorectal cancers [63]; this translocation product markedly stimulates Wnt/β-catenin signalling. RSPO2 and RSPO3 translocations appear to be mutually exclusive with APC mutations, consistent with the idea that the Wnt/β–catenin pathway needs to be activated one way or another in colorectal cancers.

**Figure 2:**
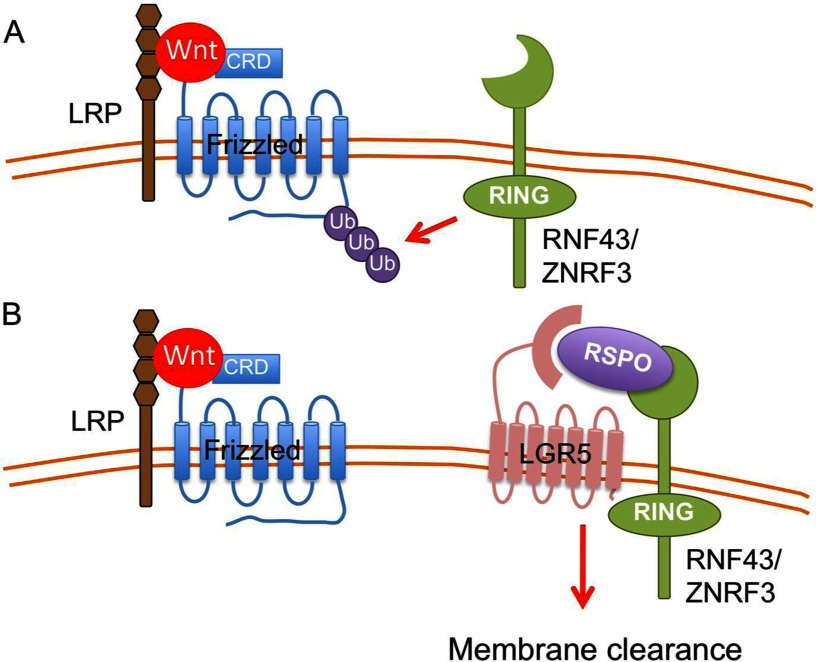
Surface level of Frizzled receptor is regulated by R-Spondin and RNF43/ZNRF3 (**A**) In the absence of RSPO (R-Spondin), negative regulator E3 ubiquitin ligase RNF43 and ZNRF3 ubiquitinate Frizzled and lead to receptor degradation. (**B**) When RSPO is present, it binds to the ectodomain of RNF43/ZNRF3 and forms a complex through association with LGR5 ectodomain. This results the membrane clearance of RNF43/ZNRF3 and increases the receptor stability.

The crystal structure of R-Spondin binding to the ectodomains of LGR5, RNF43 and ZNRF3 has been solved recently [64,65]. Together with other structural studies, this supports the model that R-Spondin is bridging LGR5 and RNF43/ZNRF3 through its Furin domains to form a ternary complex [66–68] (Figure 2). It has also been reported that binding of R-Spondin stabilizes ZNRF3 dimerization [64]. In *Drosophila*, it has been suggested that UBPY could be the deubiquitinating enzyme for Frizzled [69] and might reverse the effects of RNF43/ZNRF3. Although the counterpart has not been studied comprehensively in the mammalian system, the mutations of RNF43, ZNRF3 and RSPO2/3 in cancer supports the importance of this mechanism to fine-tune cell responsiveness to Wnt.

### Dedicated Wnt production and secretion pathway

The downstream Wnt signalling cascades have been extensively studied in the past three decades. More recently, the study of the production and secretion of Wnt proteins has garnered increased attention as well.

### Wnt ligand post-translational modification

Protein maturation and proper folding require several cellular processes after its translation. Wnt proteins contain 22 or 24 conserved cysteine residues that are important for proper folding or oligomerization of the proteins through the formation of 11 or 12 intra–molecular disulphide bonds [70,71]. Post-translation modifications of the murine WNT3A protein are the most extensively characterized [72,73]. Although the primary sequence of WNT3A is hydrophilic, the endogenous protein is hydrophobic and partitions in the detergent-phase in the Triton X-114 phase separation assay [74]. While initial studies suggested that cys-77 (Cys^55^ in XWnt8) was lipid modified, subsequent investigation supports the conclusion that Wnts contains only O-linked palmitoleate, on a conserved serine corresponding to Ser^209^ of WNT3A [70,72,75]. Desaturation of the palmitate to palmitoleate occurs via SCD (stearoyl CoA desaturase) prior to its attachment to the Wnts [75].

### Wnt and receptor complex crystal structures

**Figure 3:**
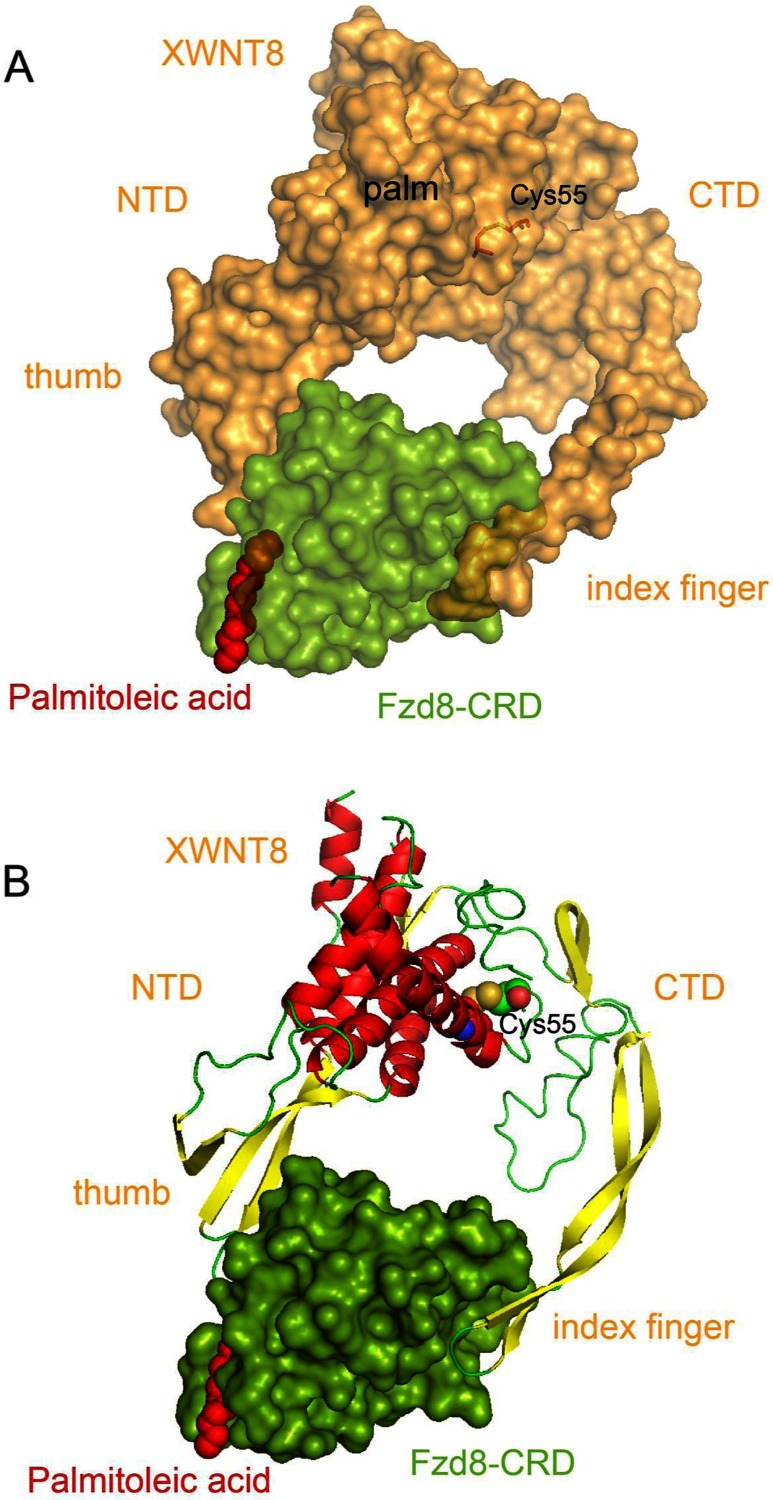
Structure of XWNT8 complexed with Fzd8-CRD (**A**) Surface representation of XWNT8 (yellow) and Fzd8-CRD (green). (**B**) Ribbon model of XWNT8, with red α-helix and yellow β-sheets secondary structures. The palmitoleic acid at Ser^187^ (red) is positioned at the tip of the ‘thumb’ of XWNT8. The ‘index finger’ of XWNT8 forms the second interaction site with Fzd8–CRD. The Cys^55^ originally proposed to be acylated instead forms an intramolecular disulphide bond with Cys^66^ [shown in sticks in the palm region in (A), and spheres in (B)]. The structure was obtained from Protein Data Bank, ID: 4F0A. The images were generated using MacPyMOL. NTD, N-terminal domain. CTD, C-terminal domain.

The hydrophobic nature of Wnt made its purification difficult, and its crystallization near impossible, until *Xenopus* WNT8 (XWNT8) was co-expressed, co-purified and co-crystallized with the Wnt-binding CRD of Fzd8 [70]. The structure of XWNT8 comprises two subdomains, an NTD (N-terminal domain) and a CTD (C-terminal domain), connected by a flexible linker region. Overall, the structure resembles a striking ‘thumb and index finger’ grasping the Fzd8–CRD at two sites, with the palmitoleate extending from the thumb to increase the interaction with Fzd8. Variation in the sequence of various Wnts and Frizzled in the interaction domains is likely to determine Wnt–Fzd-binding specificity (Figure 3). The mono-unsaturation of the palmitoleate causes a kink in the fatty acid chain and this specific structural feature may also play a role in the interaction of Wnts with both Frizzled and the carrier protein WLS (Wntless) [76].

Notably, the palmitoleated serine residue is conserved in all Wnt members across different species except in WntD, a *Drosophila* Wnt family member that does not undergo lipid modification [77]. Comparison of the crystal structure of a WntD fragment with the XWNT8 structure suggest that the positively-charged linker is responsible for interaction with the negative-charged LRP extracellular repeat 3 propeller domain [78,79].

### Porcupine/PORCN (protein Porcupine) is the acyltransferase for Wnt

The ER resident PORCN is both necessary and sufficient to catalyse the lipid modification of Wnts [80–84]. Initially identified as a segment polarity gene in *Drosophila*, PORCN belongs to the superfamily of MBOATs (membrane-bound O-acyltransferases) [85]. Since Wnt signalling is critical for embryonic development, it is not surprising that mutations in the pathway genes have been reported in many human genetic diseases [1]. Inherited and post-zygotic mutations in PORCN, an X-linked gene, cause FDH (focal dermal hypoplasia, also known as Goltz syndrome), a disease that predominantly affects females [86,87]. Global ablation of *Porcn* in mice is embryonic lethal, as the embryo fails to complete gastrulation [88]. This explains the female-specific inheritance of FDH., Mutant males suffer embryonic lethality, whereas mutant females survive and exhibit variable clinical manifestations as a result of random X-chromosome inactivation. To study PORCN developmental functions after gastrulation, conditional knockout mice have been generated using the epiblast-active *Sox2-Cre* driver [89]. Female heterozygous *Porcn* mutant (PORCN^Δ/+^) mice showed a range of abnormalities in limbs and dermis that resemble the human FDH disease [89]. These findings provide strong evidence of the aetiology of human FDH, suggesting the tissue-specific failure of Wnt ligand secretion as the cause of the disease.

PORCN appears to be the only acyl-transferase capable of modifying Wnts. Using specific zinc-finger nuclease technology, the single *PORCN* allele in the X-chromosome was inactivated in male HT1080 fibrosarcoma cells [83,84]. In these PORCN-null HT1080 cells, all 19 human Wnts lose activity, and re-expression of PORCN was required for either Wnt/β-catenin signalling, Wnt-induced Dvl-2 phosphorylation, or Wnt–WLS binding. Although WNT5B did not have any known signalling activity in these assays, it still required PORCN for binding to WLS. This underscores the importance of PORCN as the single acyltransferase in the regulation of Wnt ligand secretion and Wnt signalling. Using this cell line, our group studied the activity of the PORCN mutants found in human FDH patients. Consistent with the notion that PORCN is exquisitely required for Wnt signalling, mutants with only subtle reduction in the enzyme activity are associated with the severe form of the disease [84]. Interestingly, knockdown of PORCN in some cell lines produces a slow growth phenotype that is rescued by catalytically inactive PORCN, suggesting PORCN has additional functions in addition to acylation of Wnts in some cancer cells [90].

Studies with PORCN knockouts are consistent with prior studies of Wnt, β-catenin and LRP5/6 germline mouse knockouts that show that cell-autonomous Wnt signalling is not essential for the proliferation of embryonic stem cells. While several Wnt mRNAs are detectable in blastocysts, knockout of PORCN, WLS and the double knockout of LRP5 and LRP6 all demonstrate that the embryonic requirement for Wnt protein function only appears at gastrulation, and then later in a subset of tissues in the adult mouse. [62,88,89,91–95]. Studies with potent small molecule PORCN inhibitors further indicate that Wnt secretion is not required for the proliferation of most cultured cell lines [96].

### Wntless/WLS is the carrier protein for Wnt

A second essential Wnt secretion protein was identified through screening in the *Drosophila* system. The multipass transmembrane protein Wntless (WLS) is a Wnt-sorting receptor that carries Wnt out to the cell surface for release [97–99]. It is also highly conserved from Cnidarians to human [100].

In *Xenopus*, WLS is required for XWNT4 secretion and morpholino-mediated knockdown of WLS resulted in eye development defects [101]. In mice, *Wls* knockout is embryonic lethal similar to the *Porcn* knockout with failure of mesoderm induction and gastrulation [95]. It has been reported that in the *Drosophila* larval neuromuscular junction, Wingless (Wg, the homologue of Wnt1 in the fly) is secreted in the WLS-containing exosome-like vesicles from the presynaptic neurons [102]. Jin et al. further identified the mu-opioid receptor as a WLS-binding partner, which sequesters WLS on the cell surface in the presence of an opioid agonist, thus inhibiting Wnt protein secretion [103]. These data suggest that the regulation of Wnt secretion by WLS is complex.

Several conditional *Wls* knockout mouse models have been developed that highlight the essential role of WLS in development. When the floxed *Wls* allele is excised by *Wnt1-Cre*, the resulting mice exhibit a phenotype similar to the *Wnt1* null mice, with developmental abnormalities in the midbrain and hindbrain [104]. Recently, genome-wide association studies identified intronic SNPs (single-nucleotide polymorphisms) in the WLS gene that are strongly associated with reduced bone mineral density [105]. Consistent with the association study, when *Wls* was specifically deleted in mature osteoblast and osteocytes, the mice developed severe low bone mass due to reduced bone formation and increased matrix resorption [106]. These mice were prone to fractures and could not survive long after birth. Wnt/β-catenin signalling is also critical in skin and hair. Accordingly, conditional deletion of *Wls* with *Keratin 14-Cre* driver results in the loss of hair follicles and infiltration of immune cells into the skin together with up-regulation of inflammation-related genes [107,108].

Unlike *PORCN*, *WLS*-coding region mutations are not reported to date in any human diseases. WLS expression levels vary widely, however, and are elevated in some cancers, including astrocytic gliomas and glioblastoma cell lines [109]. On the other hand, *WLS* expression is reduced in melanoma tumours as compared to the normal skin and benign lesions [110]. Knockdown of *WLS* in melanoma cell lines increased cell proliferation *in vitro* and promoted lung metastasis in a xenograft model [110], consistent with a tumour suppressive role of Wnt/β-catenin signalling in this disease [111]. These findings further highlight the importance of cellular context in determining the role of Wnt signalling in a specific disease.

### Factors that regulate WLS trafficking-retromer complex and SNX3

At the molecular level, WLS is a cargo receptor that transports Wnt from the Golgi apparatus to the plasma membrane. The interaction of Wnt and WLS is dependent on the palmitoleation of Wnt at the serine residue mediated by PORCN [76,84,112]. Upon dissociation from Wnts at the cell surface, WLS is recycled via clathrin-mediated endocytosis. The tyrosine-containing motif YXXΦ (where Φ is any hydrophobic amino acid) in the third intracellular loop of WLS is required for its endocytosis from the cell surface, as WLS accumulates on the plasma membrane when the corresponding Y425 is mutated [113,114]. Following internalization, the retromer complex drives retrieval of WLS from the endosomes to the *trans*-Golgi network [115–117]. The involvement of the retromer complex in Wnt signalling was first discovered in *Caenorhabditis elegans*, as the *vps-35* mutant produced an EGL-20/Wnt-defect phenotype [118]. In the budding yeast *Saccharomyces cerevisiae*, the retromer complex is composed of two subcomplexes, cargo-selection subunits consisting of Vps35p, Vps29p and Vps26p; and structure subunits, consisting of Vps5p and Vps17p dimer [119]. WLS interacts with Vps35 in immunoprecipitation experiments [115]. In the absence of Vps35, WLS accumulates instead in multi-vesicular bodies, as shown by electron microscopy [116]. Therefore the improper recycling of WLS results in the reduced levels of the protein in Vps35-depleted cells [115]. To date, the sorting signal on WLS that enables cargo-selection by Vps35 has not been identified.

The retromer complex is associated with the endosomal membrane, probably through interaction with lipid-enriched elements found in the endocytic network [119]. SNX-1 (sorting nexin-1), the homologue of yeast Vps5p, has a Phox-homology (PX) domain that binds to phosphatidylinositol 3,5-bisphosphate (Ptd(3,5)P_2_) commonly found on the early endosomes [120]. Interestingly, MTM-6 and MTM-9, members of the myotubularin family, have also been implicated in the MIG-14/WLS trafficking in *C. elegans* [121]. Myotubularin is a lipid phosphatase that dephosphorylates phosphatidylinositol 3-phosphate (Ptd3P) and Ptd(3,5)P_2_, which could potentially affect endosomal trafficking. However, in contrast to the conventional SNX1-/SNX-2 and SNX-5/SNX-6 (homologous to yeast Vps17p) dependent retromer pathway, an unrelated SNX-3 appears to participate in the retrograde transport of WLS [122,123]. SNX-3 co-localizes with WLS and can co-immunoprecipitate with the cargo selective subunits, Vps35 and Vps26, of the retromer complex [122]. Conventional SNXs possess a BAR (Bin-Amphiphysin-Rvs) domain, which drives the membrane curvature and cargo segregation into the tubular structure [124]. However, it is still unknown how SNX-3, which lacks the BAR domain, regulates the recycling of WLS.

When retromer complex function is partially disrupted in *C. elegans* by a *vps29* mutant, the retrieval of WLS/MIG-14 can be restored by blocking endosomal maturation. Moreover, WLS/MIG-14 that accumulates in the late endosomes can be partially retrieved through the classical SNX–BAR-dependent pathway in *vps29* mutant, but not in *snx-3* mutant [125]. The authors therefore proposed that both the spatial distribution of SNX3 (in early endosomes) and SNX–BAR (in early-to-late and late endosomes), as well as cargo-specific mechanisms contributes to the retromer-dependent retrieval of WLS [125].

### p24 protein family members in Wnt secretion

The transport of Wnt from the ER (endoplasmic reticulum) to the Golgi complex relies on a sequence of protein-sorting events in the early secretory pathway. Specific COP (coat protein) complexes are involved in this bidirectional trafficking of proteins. Generally, COPII mediates the anterograde transport from the ER to the Golgi, whereas COPI generates the retrograde trafficking from the Golgi back to the ER. However, there is also evidence showing that anterograde-directed cargos are present in a distinct population of COPI vesicles [126].

Genome-wide RNAi screening in *Drosophila* revealed the a role for the p24 family of proteins in Wnt secretion [127,128]. p24 proteins are components of the COP vesicles, and have previously been implicated in the regulation of cargo sorting and trafficking [129]. In mammals, there are at least eleven p24 family members. It has been suggested that p24 proteins are involved in the ER exit of GPI (glycosylphosphatidylinositol)-anchored proteins in yeast [130,131]. Similar roles of p24 proteins have also been proposed by Port et al. [128] as to facilitate Wg export from the ER in *Drosophila*. However, there is only limited evidence supporting the role of p24 in mammalian Wnt secretion and the redundancy of p24 family members complicates their study.

### Retrograde trafficking to the ER

**Figure 4:**
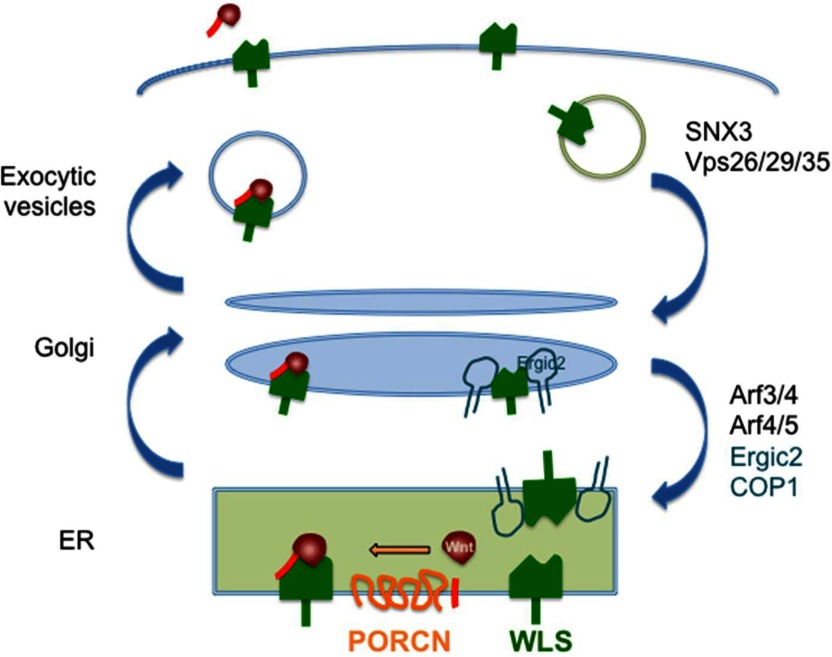
The Wnt secretion pathway Newly synthesized Wnts are palmitoleated (red line) by PORCN in the ER, facilitating binding to the specialized transporter WLS (green). WLS carries Wnts to the plasma membrane for release after acidification in exocytic vesicles [76]. WLS is recycled via clathrin-coated vesicles and the retromer complex to the Golgi, and then to the ER using COP1 vesicles and ERGIC2 [114]. Alternative routes for Wnt exit also exist (see text).

WLS cycles from the Golgi to the plasma membrane and then back to the Golgi via the retromer complex after endocytosis, but it is not known how the palmitoleated Wnts in the ER meet up with WLS in the Golgi. A high-affinity monoclonal antibody against human WLS was developed that specifically recognizes endogenous WLS [76]. Using a diverse set of approaches, we found that after endogenous WLS cycles to the cell surface, it then returns to the ER through the Golgi [114]. WLS contains a unique ER targeting sequence at its C-terminus that is critical for both its ER recycling and biological function. C-terminal deletion, point mutation or epitope tagging inhibits WLS ER localization and activity in Wnt/β-catenin signalling. In addition, Golgi to ER retrograde recycling of WLS requires the COPI transport machinery involving the function of the small GTPase ARF as well as ERGIC2 [114]. These findings solve the problem of how to get lipid-modified Wnts out of the ER. Whether release of WLS from the ER is itself regulated remains to be determined, it is notable that WLS is the first example of an integral membrane secretory protein that cycle from ER to the PM and back to the ER. The Wnt secretion pathway is shown in Figure 4.

### Wnt extracellular trafficking

As noted above, Wnt secretion is a complex process involving specialized cellular machinery that controls the export of Wnt. In addition, the presence of a fatty acid chain on Wnts also increases their hydrophobicity, posing challenges to the spreading of Wnt in the extracellular matrix that is required to exert signalling as a morphogen. Although this process is still poorly understood, a few models have been proposed to better account for Wnt extracellular trafficking. Some examples of these models are described below.

In *Drosophila* wing imaginal disc, filopodia-like cellular extensions called cytonemes have been observed in outlying (signal receiving) cells oriented towards the disc centre (signal secreting) cells [132]. This type of cell–cell contact might mediate the intermediate-range transport of Wnt, although no direct evidence has been reported so far.

HSPGs (heparan sulphate proteoglycans) are another group of molecules that mediate the lateral diffusion of Wnt proteins. Glypican Dally and Dlp (Dally-like proteins) are involved in gradient formation by transferring Wg to neighbouring cells, thus inhibiting the binding of Wg to its local receptor for signalling [133].

Alternatively, a complex Wnt-containing structure might be formed, which shields the lipids attached to the protein from the extracellular environment. Self-aggregation into a micelle might reduce the hydrophobicity of the Wnt molecule. However, monounsaturated palmitoleate, unlike palmitate, might not favour micelle formation. This mechanism has been reported for the Hedgehog protein, another palmitoylated signalling protein that requires long-range signalling during development [134]. It has been suggested that Wnts can bind to lipoprotein particles such as lipophorin in *Drosophila* to facilitate their movement [135,136]. Secreted Swim (Wingless-interacting molecule), a member of the lipocalin family proteins that bind lipids in a tertiary barrel-like structure, has been identified recently to bind *Drosophila* Wg with high affinity [137]. More importantly, Swim is both necessary and sufficient to solubilize purified Wg proteins *in vitro* [137].

Recent studies from the Budnik laboratory provide strong evidence that Wg is transported across synapses in WLS-containing exosome-like vesicles at the larval NMJ (neuromuscular junction) in *Drosophila* [102]. The release of WLS-containing exosomes at the synaptic boutons seems to require the small GTPase Rab11 and Syntaxin 1A [138]. In cultured *Drosophila* S2 cells, Wg is also secreted on exosome-like vesicles, which depends on the Rab11 function as well [139]. However, the Wg gradient formation in the wing imaginal disc is not affected when Rab11 is depleted by RNAi in the tissue [139]. In mammalian cells, Wnt proteins can be secreted on exosomes derived from endosomal compartments in a process that requires the R-SNARE (SNAP receptor) protein Ykt6 [140]. Exosome-mediated Wnt signalling may play a role in cell migration and wound healing [141–143].

## DRUG DISCOVERY IN THE WNT FIELD

### Wnt signalling and cancers

Since Wnt signalling controls cell growth, differentiation and migration, the aberrant activation of Wnt signalling can lead to uncontrolled cell growth and contribute to tumourigenesis. The molecular evidence that Wnt is involved in both embryogenesis and cancer development demonstrates the convergence for these two fields [144].

A well-known example that links the Wnt pathway with cancer development is a hereditary disease condition known as FAP (familial adenomatous polyposis). FAP patients inherit a mutated copy of the APC gene, and the second APC allele is often lost later in life [145–147]. These mutations found on APC are usually nonsense or frame shift mutations, which generate truncated forms of APC. The loss-of-heterozygosity phenomenon observed in the disease suggests that APC may exert a potential tumour suppressive function. Affected individuals usually develop multiple adenomatous polyps in the colon at a relatively early age. These polyps can transform into carcinomas upon acquisition of additional mutations. Besides mutations in APC, inactivating mutations in Axin2 as well as gain-of-function mutations in β-catenin have also been found in colon cancer patients, albeit with relatively low frequency [148–150]. The Cancer Genome Atlas Network in 2012 reported Wnt/β-catenin pathway mutations in up to 93% of colorectal cancers, and that was prior to the identification of RPSO2/3 translocations and RNF43/ZNRF3 mutations in the Wnt pathway [151]. Stabilizing mutations in β-catenin are commonly found in gastric, liver and kidney cancers as well.

### Current advances in Wnt pathway inhibitors

Given the therapeutic potential in the treatment of Wnt-related cancers, there are diverse attempts to target the Wnt pathway with small molecule inhibitors. The identification of valid drug targets is challenging, although an increasing number of proof-of-concepts studies in animal models have been reported and recently reviewed [152]. The effective therapies can be divided into downstream inhibitors, those that target β-catenin abundance and/or activity [153,154], and upstream inhibitors, those that target Wnt production or Wnt interaction with extracellular receptors.

Downstream inhibitors include tankyrase inhibitors. This class of compounds stabilizes Axin protein and thus promotes the function of the destruction complex. The earliest examples of these include IWR-1 [155] and XAV939 [156]. Both XAV939 and IWR are inhibitors of the poly-ADP-ribosylating enzyme tankryase, which facilitates Axin turnover by PARsylation-mediated ubiquitination and proteosomal degradation [156].

ICG-001 inhibits the interaction of β-catenin with the histone acetyltransferase and transcriptional co-activator CBP, hence blocking transcriptional activation of a subset of β-catenin regulated genes. A related compound, PRI-724, is now in clinical trials for acute myeloid leukaemia (clinicaltrials.gov NCT01606579) and advanced solid tumours (clinicaltrials.gov NCT01302405) [154].

Inhibition of upstream Wnt activity can be achieved by small molecule inhibition of PORCN activity [76]. Novartis has developed a PORCN inhibitor LGK974, which entered Phase I clinical trial in 2012 (clinicaltrials.gov NCT01351103). This compound, as well as another related compound C59, showed efficacy in Wnt-driven tumours in mouse models at well-tolerated doses [96,157]. Importantly, pancreatic ductal cancer cell lines with inactivating mutations in RNF43 are sensitive to LGK974, providing a genetic biomarker for responsive patients [59]. A second promising approach to upstream inhibition of Wnt signalling is the use of monoclonal antibodies such as vantictumab that bind to the extracellular domain of Frizzled receptors and prevent the interactions with Wnts [158] (clinicaltrials.gov NCT02005315 and others). One concern in all these studies is whether effective Wnt pathway inhibition will have significant side effects due to inhibition of essential Wnt-driven processes such as bone formation.

### Remaining questions in the field

As we learn more about Wnt biology, new questions continue to arise. There continues to be controversy about how Wnts are transported outside the cell. Lipoproteins and argosomes were identified first, while the exosome model is gaining traction. More work is needed to define the molecular origin of the exosomes and how Wnts are incorporated into these vesicles. Even the concept of Wg as a secreted morphogen in the *Drosophila* wing imaginal disc has been challenged [159]. When a membrane-tethered *Wg* was expressed in *Drosophila* in the absence of the endogenous alleles, the resulting flies were viable with almost normal wing size [160]. This implies that Wg spreading across distance is not necessary for patterning and growth in *Drosophila*. The authors reasoned that the *Wg* transcription is active during the initial stage of wing patterning, and only restricted to the dorsoventral boundary later on. Nevertheless, the expression of target genes such as *vestigial* and *Distal-less* persists at a lower level. This partially explains the slow growth phenotype of flies with membrane-bound *Wg* [160]. This study prompts us to question some long-held assumptions of the Wg morphogen model.

Limited studies have been done to investigate the Wnt secretory pathway in a polarized cell system, which is more relevant to normal physiology. At least for WNT3A and WNT11, glycosylation seems to regulate whether Wnt is secreted from the apical or basolateral surface of polarized epithelial cells [161]. However, the role of WLS in this polarized trafficking is less well defined.

In the context of the tissue or organ, it will be important to identify the source of the Wnt. For example, Paneth cells at the bottom of the small intestine crypts were reported to be the critical Wnt-producing cells that support the *LGR5*-positive CBC (crypt base columnar) cells [162], based on *ex vivo* data. However, several recent *in vivo* studies call this model into question. In the absence of Paneth cells, or if epithelial Wnt secretion is blocked by knockout of PORCN or WLS, intestinal stem cells are still functional *in vivo* [62,163,164]. In fact, our group found that underlying stromal intestinal myofibroblasts robustly support Wnt-deficient Lgr5+ intestinal stem cells in organoid formation and in this setting, organoids no longer require RSPO supplementation. Importantly, these myofibroblasts express multiple Wnts, and high levels of RSPO3. Hence, as in many other stem cell niches, critical trophic factors derive from the stroma rather than the proliferating compartment.

## SUMMARY

Because of their central role in both development and disease, only a few signalling pathways have received as much scrutiny over the years as Wnt signalling. This intensive study continues to yield surprising insights and unexpected mechanisms. Our ability to target the pathway therapeutically is gaining in sophistication, and the number of clinical trials ongoing suggests we will learn in the coming years if therapeutic intervention in the Wnt pathway is safe and effective.
